# MiR-19b-3p regulated by BC002059/ABHD10 axis promotes cell apoptosis in myocardial infarction

**DOI:** 10.1186/s13062-022-00333-x

**Published:** 2022-08-18

**Authors:** Bihong Liao, Shaohong Dong, Zhenglei Xu, Fei Gao, Suihao Zhang, Ruijuan Liang

**Affiliations:** 1 Department of Cardiology, Shenzhen People’s Hospital, Second Clinical Medical College of Jinan University, 1017 Dongmen North Road, Luohu District, Shenzhen, 518000 Guangdong China; 2 Department of Gastroenterology, Shenzhen People’s Hospital, Second Clinical Medical College of Jinan University, 1017 Dongmen North Road, Luohu District, Shenzhen, 518000 Guangdong China

**Keywords:** Myocardial infarction, Apoptosis, miR-19b-3p, BC002059/ABHD10 axis

## Abstract

**Background:**

Recently, microRNAs (miRNAs), have been extensively investigated in diseases. The upregulated expression of miR-19b-3p has been validated in patients with hypertrophic cardiomyopathy. Nonetheless, it regulatory mechanism in myocardial infarction (MI) is still unclear.

**Purpose:**

This research aimed to investigate the role and molecular regulation mechanism of miR-19b-3p in MI.

**Methods:**

QRT-PCR and western blot assays measured RNA and protein expression. Cell apoptosis were tested by flow cytometry and TUNEL assays. Cell viability was measured by trypan blue staining method. RIP and luciferase report assays examined gene interaction. The assays were performed under hypoxia condition.

**Results:**

MiR-19b-3p was highly expressed in myocardial cell line H9C2, primary cardiomyocytes, and tissues from MI mouse model. MiR-19b-3p inhibition suppressed the apoptosis of cardiomyocytes. BC002059 could up-regulate ABHD10 through sequestering miR-19b-3p. BC002059 upregulation was observed to repress cell apoptosis. Rescue experiments demonstrated that miR-19b-3p overexpression abrogated the suppressive impact of BC002059 on the apoptosis of MI cells, and infarct size, area at risk as well as CK-MB and LDH release of MI mouse model tissues, which was further abolished via ABHD10 increment.

**Conclusion:**

MiR-19b-3p regulated by BC002059/ABHD10 axis promotes cell apoptosis in MI, which might provide a novel perspective for MI alleviation research.

## Background

Myocardial infarction (MI) is identified as the main death-related disease in most industrialized nations, featuring with cardiomyocyte death [1]. Though reperfusion and drug treatment greatly alleviate MI, poor left ventricular remodeling after MI is still the most common cause of heart failure [2, 3]. In MI and secondary heart failure, myocardial structural changes include myocardial apoptosis, extracellular matrix protein increase, myocardial fibrosis, and myocardial hypertrophy [4]. In the past few decades, researchers have paid much attention to figure out the method about stimulating infarcted heart regeneration. Nonetheless, there are still many obstacles to overcome before finding out efficient treatment.

Recent research has demonstrated the regulatory role of non-coding RNAs (ncRNAs) in microRNA (miRNAs)-involved network [5]. MiRNAs belong to short RNAs containing about 22 nucleotides. Existing evidence suggests multiple biological mechanisms involving miRNAs in the occurrence and development of diseases. For example, miR-92a alleviates the damage on pancreatic B-cell function via targeting KLF2 in diabetes mellitus [6]. MiR-126 hinders the blood thrombogenicity via targeting tissue factor in diabetes mellitus [7]. MiR-21 plays the role in weakening lipopolysaccharide-induced lipid accumulation and relieving inflammatory response in cerebrovascular disease [8]. MiR-744/TGF-β1 axis modulates liver cirrhosis [9]. Moreover, miR-29a-3p serves as a plasma biomarker for diagnosing and monitoring tuberculosis [10].

The regulation role of miRNAs in cardiac diseases has recently attracted growing attention. For example, miR-133 exerts regulatory function in cardiac diseases [11]. MiR-206 predicts the severity of pulmonary hypertension in left heart disease patients [12]. Simvastatin treatment suppresses myocardium apoptosis in noncoronary artery cardiac surgery via targeting miR-15a-5p [13]. In addition, the expression of miR-19b-3p has been validated to be upregulated in hypertrophic cardiomyopathy patients [14]. Nevertheless, the molecular regulation mechanism of miR-19b-3p in MI remains to be studied. Additionally, myocardial apoptosis plays a vital part in the etiopathogenesis and development of heart failure induced by MI [15]. Cardiomyocytes apoptosis has become the focal point in MI studies. For instance, miR-124 inhibitor suppresses MI-caused cardiomyocyte apoptosis by targeting STAT3 [16]. Long non-coding RNA ZFAS1 modulates apoptosis of cardiomyocytes caused by acute MI through regulating the expression of miR-150 and CRP [17].

In our research, we targeted at exploring the function of miR-19b-3p in cell apoptosis in MI as well as its molecular mechanism, which may provide some novel directions for studying MI-related molecular mechanisms.

## Results

### Hypoxia induces apoptosis of H9C2 cells and primary cardiomyocytes

**Fig. 1:**
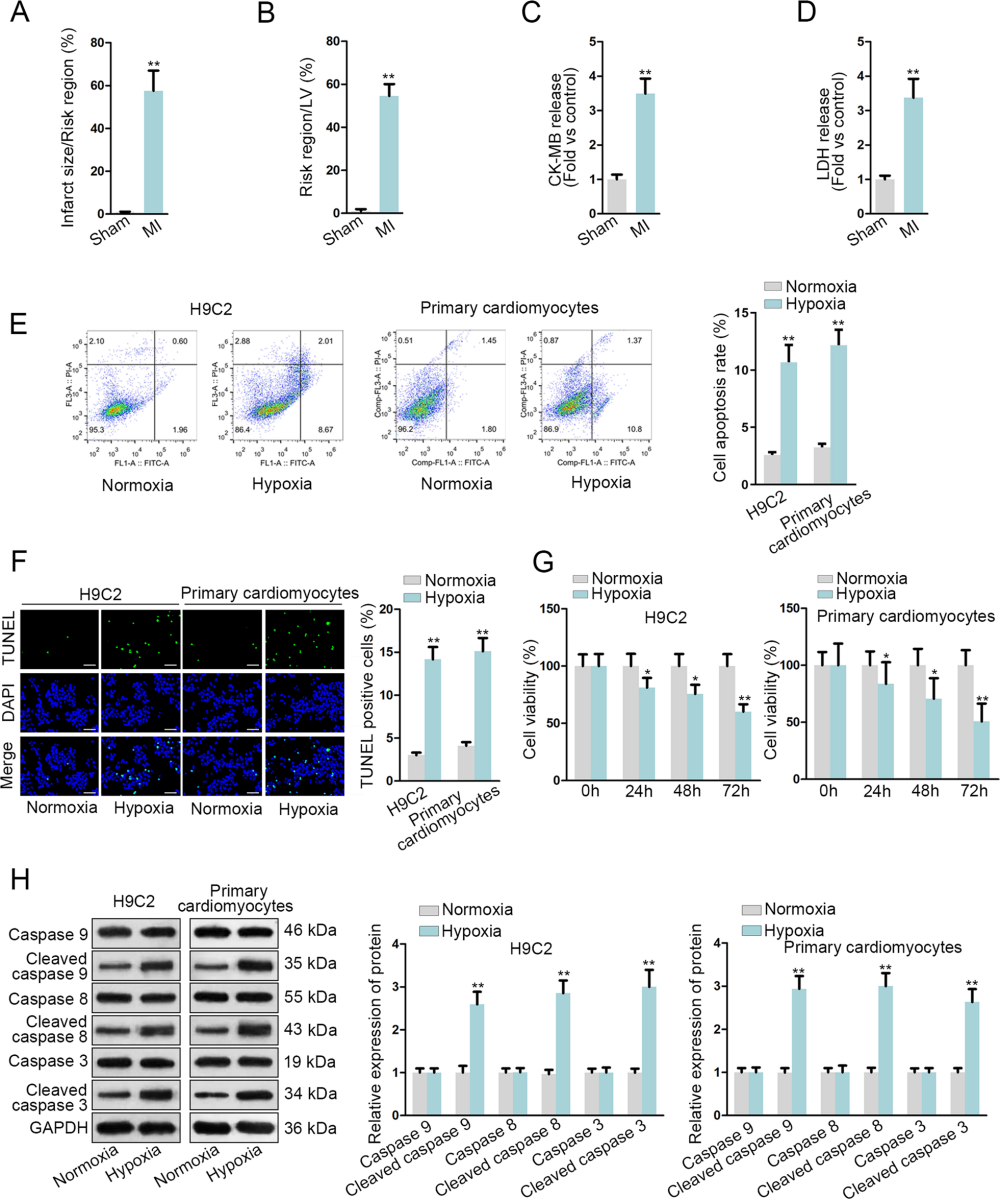
Hypoxia induces apoptosis of H9C2 cells and primary cardiomyocytes. **A** The percentage of infarct size/Risk region, **B** that of Risk region/LV **C** serum CK-MB release and **D** LDH release were detected to evaluate the effectiveness of established mouse MI model. **E**, **F** Cell apoptosis was examined through flow cytometry and TUNEL (bar value = 1:100 μm) assays. **G** Cell viability was detected through trypan blue staining assay. **H** Apoptosis-related protein levels were detected through western blot assay. *P < 0.05, **P < 0.01

Firstly, we constructed mouse MI model and sham group (sham-operated mice). Next, myocardial infarct size/risk region and risk region/left ventricle (LV), as well as CK-MB and LDH release, two biochemical markers of myocardial cell necrosis, were detected in the two groups. A remarkable increase of infarct size/risk region, risk region/LV, CK-MB and LDH release was noticed in MI mice (Fig. 1A–D), signifying the successful construction of the mouse MI model. Furthermore, according to the data collected from flow cytometry analysis and Terminal-deoxynucleoitidyl transferase mediated nick end labeling (TUNEL) assays, the apoptotic capability of H9C2 cells and primary cardiomyocytes was stimulated by hypoxia treatment (Fig. 1E, F). In addition, trypan blue staining detected cell viability of H9C2 cells and primary cardiomyocytes, and it was found that cell viability declined over time in the absence of oxygen (Fig. 1G). Moreover, western blot assay unveiled that the protein levels of cleaved caspase 9, cleaved caspase 8 and cleaved caspase 3 were elevated in cells treated with hypoxia (Fig. 1H). Overall, hypoxia strengthens the apoptotic ability of H9C2 cells and primary cardiomyocytes.

### MiR-19b-3p inhibition restricts apoptosis of H9C2 cells and primary cardiomyocytes

**Fig. 2:**
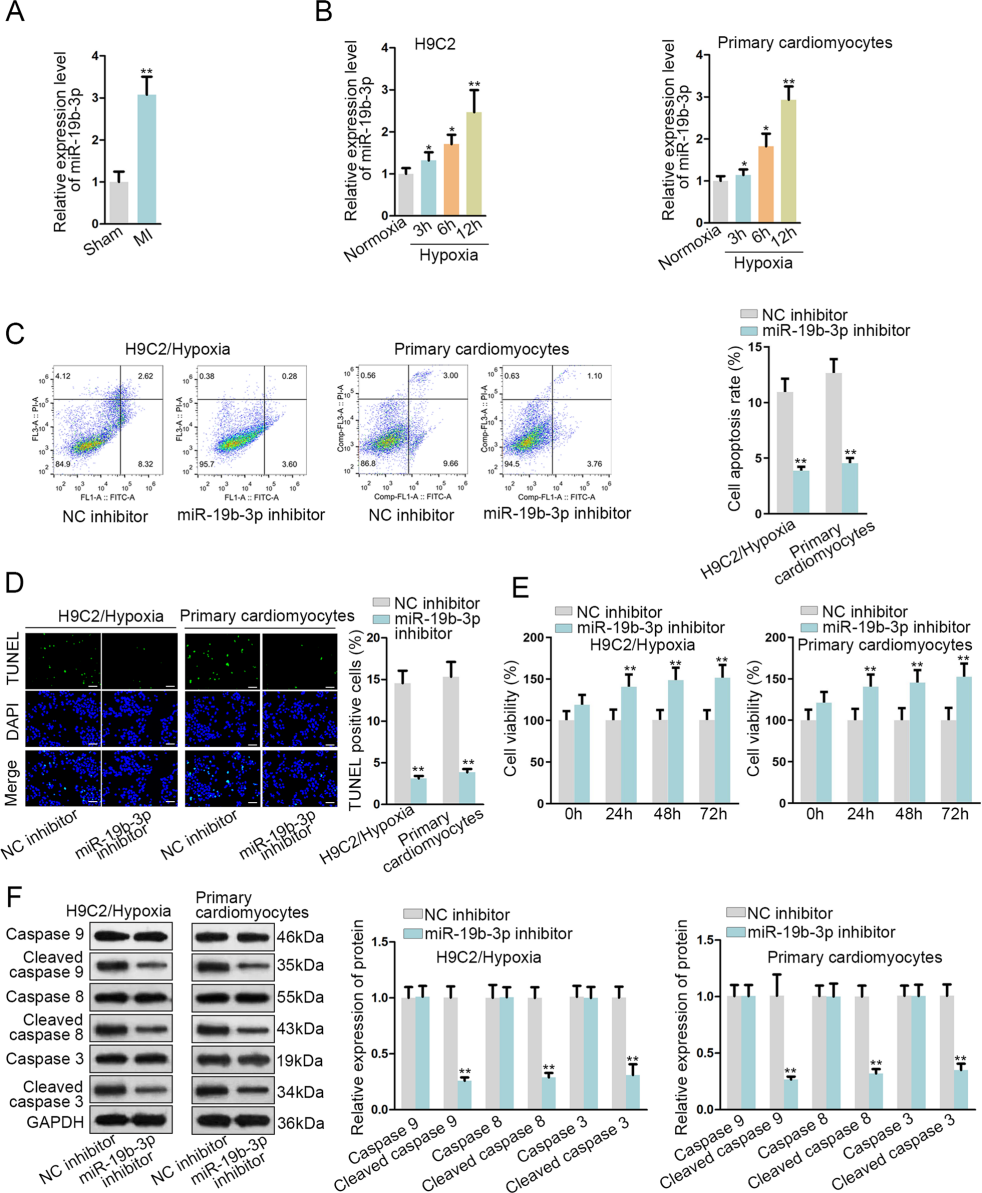
MiR-19b-3p is highly expressed in hypoxia-treated H9C2 cells and primary cardiomyocytes.** A**,** B** MiR-19b-3p expression in MI model/control group, H9C2 cells and primary cardiomyocytes upon hypoxia/normoxia treatment was detected by qRT-PCR.** C**,** D** Cell apoptosis was tested through flow cytometry analysis and TUNEL (bar value = 1:100μm) assay.** E** Cell viability was tested through trypan blue staining assay.** F** Western blot assay measured apoptosis-related protein levels. *P < 0.05, *P < 0.01

Considering the close connection between the aberrant expression of miR-19b-3p and myocardial hypertrophy patients [14], it is possible that miR-19b-3p may also affect cardiomyocyte apoptosis. Accordingly, quantitative real-time polymerase chain reaction (qRT-PCR) was performed and the obtained results revealed that miR-19b-3p was upregulated in MI model (Fig. 2 A). After hypoxia treatment, miR-19b-3p expression was elevated with time in H9C2 cells and primary cardiomyocytes (Fig. 2B). Subsequently, loss-of-function assays were implemented in H9C2 cells treated with hypoxia and primary cardiomyocytes obtained from MI model. Flow cytometry and TUNEL assays illustrated that miR-19b-3p inhibition suppressed cell apoptosis (Fig. 2C and D). Trypan blue staining assays showed cell viability of miR-19b-3p inhibitor-transfected cells was enhanced (Fig. 2E). Obtained western blots signified miR-19b-3p inhibition decreased the level of cleaved caspase 9/8/3 (Fig. 2F). In general, the expression of miR-19b-3p is upregulated in MI mice, and miR-19b-3p inhibition suppresses apoptosis of H9C2 cells and primary cardiomyocytes.

### BC002059 binds with miR-19b-3p in MI

**Fig. 3:**
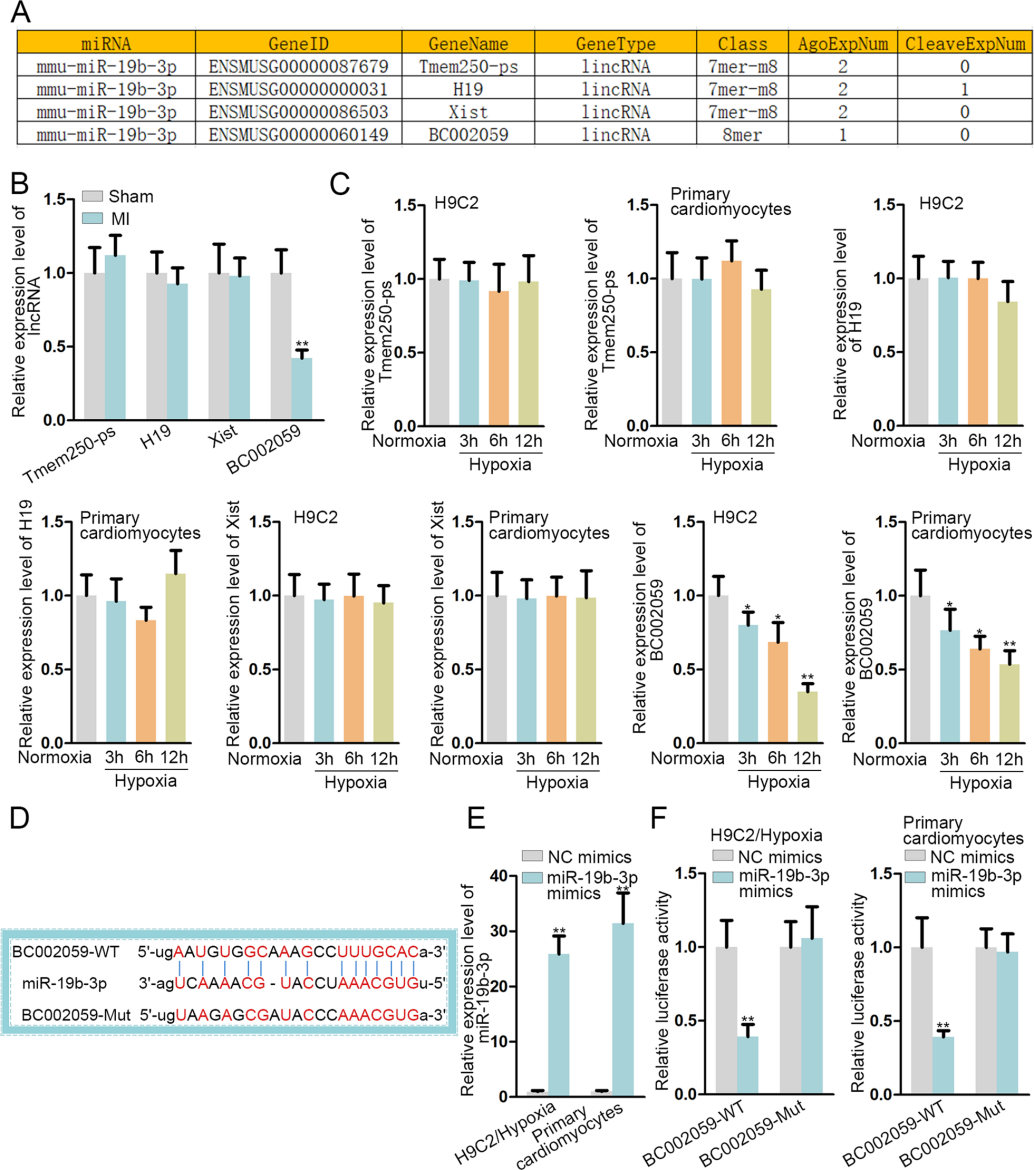
BC002059 binds with miR-19b-3p in MI. **A** The list exhibited the lncRNAs that might bind with miR-19b-3p. **B**, **C** The expression of four pre-qualified lncRNAs in MI model/control group, H9C2 cells and primary cardiomyocytes under hypoxia/normoxia condition was detected by qRT-PCR. **D** The binding sites between BC002059 and miR-19b-3p were projected on starBase. **E** The overexpression efficiency of miR-19b-3p mimics was tested through qRT-PCR. **F** Luciferase reporter assay tested the above binding sites. *P < 0.05, **P < 0.01

Considering the significance of interactions between long non-coding RNAs (lncRNAs) and miRNAs in MI [18], we aimed to find out the lncRNA that might bind with miR-19b-3p. By utilizing starBase, we screened out four lncRNAs, including Tmem250-ps, H19, Xist and BC002059 (CLIP-Data > = 2; Degradome-Data > = 0) (Fig. 3A). Thereafter, we detected the expression of the above lncRNAs in MI and Sham groups, and found only BC002059 was obviously downregulated in MI (Fig. 3B). Furthermore, after H9C2 cells and primary cardiomyocytes were subject to hypoxia treatment, the expression of Tmem250-ps, H19 and Xist had no significant change, whereas that of BC002059 was remarkably diminished (Fig. 3C). Moreover, we used bioinformatics tool (http://www.csbio.sjtu.edu.cn/bioinf/lncLocator/) and projected that BC002059 was mainly distributed in cytoplasm. The binding sequence of BC002059 and miR-19b-3p was projected by starBase (Fig. 3D). Moreover, the overexpression efficiency of miR-19b-3p mimics was validated cells (Fig. 3E). Luciferase reporter assay results manifested that miR-19b-3p augment inhibited luciferase activity of BC002059-WT (the short name for wild-type), and that of BC002059-Mut (the short name for mutant) was not affected (Fig. 3F). To summarize, BC002059 binds with miR-19b-3p in MI.

### BC002059 upregulation represses MI cell apoptosis

**Fig. 4:**
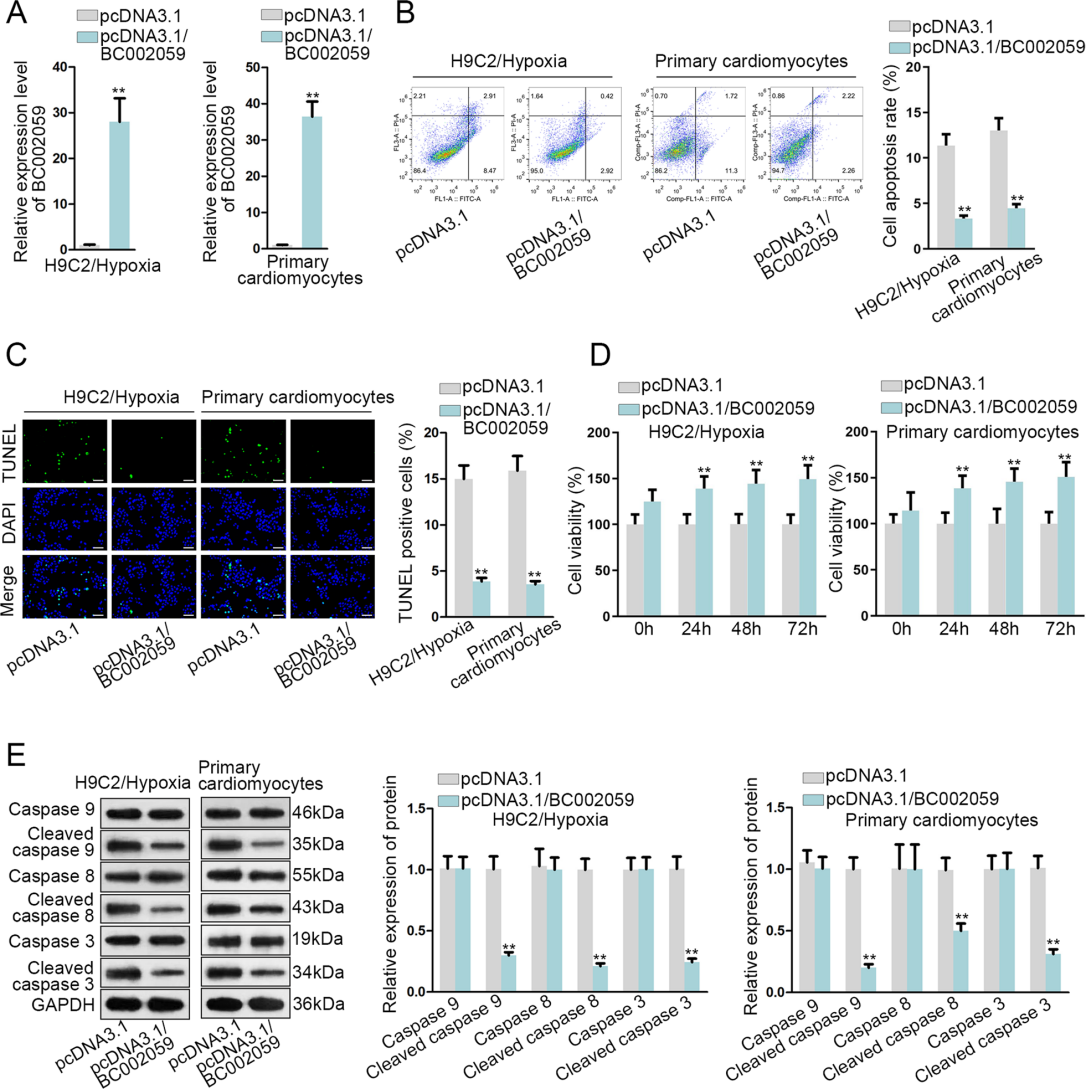
BC002059 upregulation represses MI cell apoptosis. **A** The overexpression efficiency of pcDNA3.1/BC002059 was measured through qRT-PCR. **B**, **C** Cell apoptosis was analyzed through flow cytometry and TUNEL (bar value = 1:100 μm) assays **D** Trypan blue staining assay detected cell viability. **E** Western blot assay measured apoptosis-related protein levels. **P < 0.01

In this section, the regulatory role of BC002059 in MI cells was tested. Firstly, pcDNA3.1/BC002059 plasmids were used to upregulate BC002059 (Fig. 4A). Next, based on the results of flow cytometry analysis and TUNEL assay, BC002059 overexpression suppressed cell apoptosis (Fig. 4B, C). In addition, cell viability was measured via trypan blue staining assay and it was found that cell viability was stimulated by BC002059 augment (Fig. 4D). Moreover, through western blot assay, we noticed the protein level of cleaved caspase 9/8/3 descended (Fig. 4E). Overall, BC002059 overexpression restrains MI cell apoptosis.

### ABHD10 is the downstream target of miR-19b-3p in MI

**Fig. 5:**
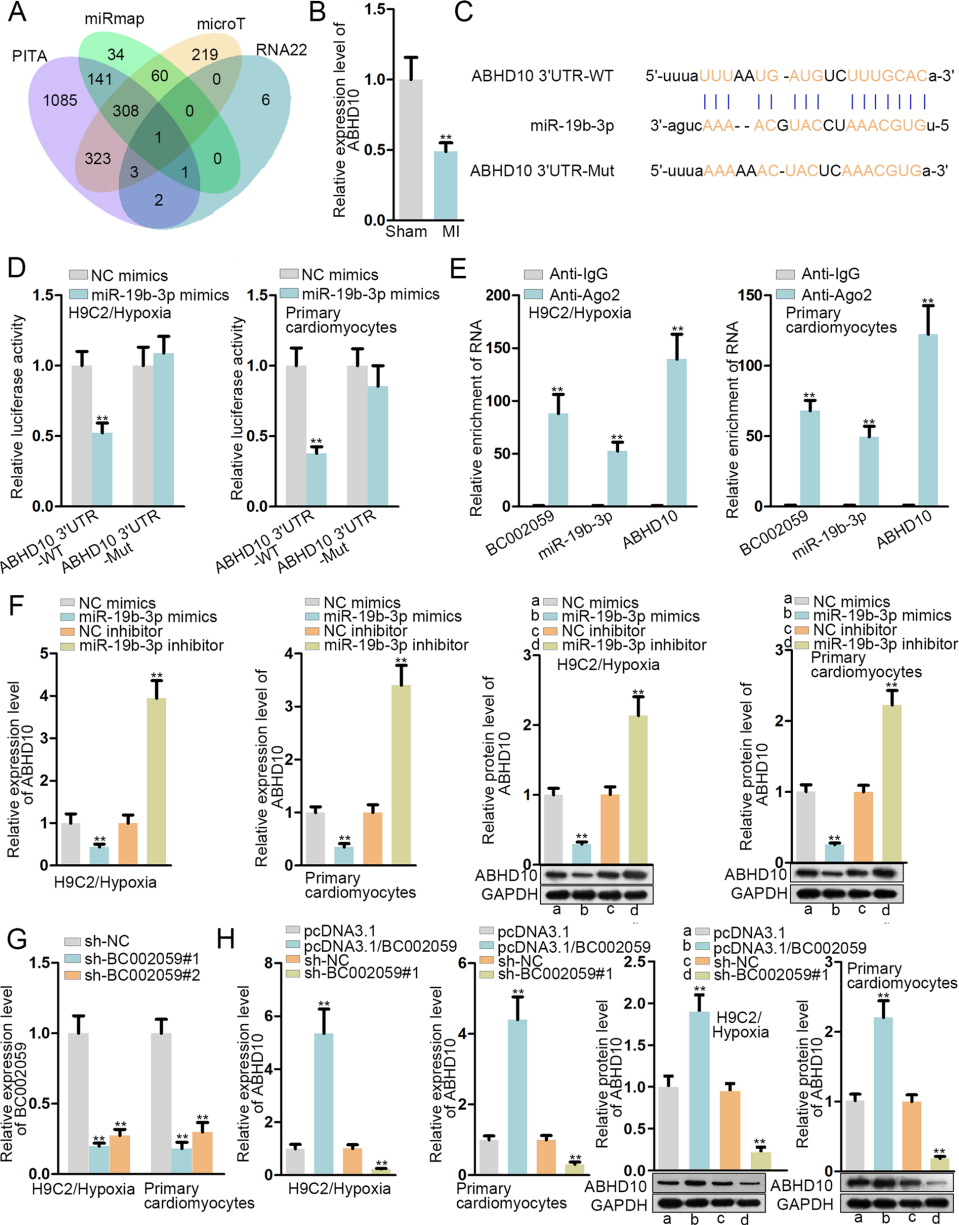
ABHD10 is the downstream target of miR-19b-3p in MI. **A** Venn diagram displayed the mRNAs which might bind with miR-19b-3p. **B** The expression of ABHD10 in MI model/control group was detected by qRT-PCR. **C** The potential binding sites between ABHD10 and miR-19b-3p were projected on starBase.** D** Luciferase reporter assay confirmed the above binding sites. **E **RIP assay measured the enrichment of BC002059, miR-19b-3p and ABHD10 in anti-Ago2 group. **F** The RNA and protein expression of ABHD10 was measured through qRT-PCR and western blot assays. **G** The knockdown efficiency of BC002059 was evaluated by qRT-PCR. **H** The RNA and protein expression of ABHD10 was measured through qRT-PCR and western blot assays. **P < 0.01

Considering the critical role of messenger RNAs (mRNAs) in regulating the development of diseases, including MI [19, 20], we decided to find out the target mRNA of miR-19b-3p. Via PITA, RNA22, miRmap and microT databases, ABHD10 was screened out to be the target mRNA of miR-19b-3p (Fig. 5A). Next qRT-PCR analysis revealed the low expression of ABHD10 in MI group (Fig. 5B). Further, starBase-predicted binding sites of miR-19b-3p and ABHD10 were exhibited in Fig. 5C. Then luciferase reporter assay confirmed the efficiency of the projected binding sequence as the luciferase activity of ABHD10 3’UTR-WT was diminished by miR-19b-3p augment (Fig. 5D). Additionally, RNA binding protein immunoprecipitation (RIP) assay uncovered the obvious enrichment of BC002059, miR-19b-3p and ABHD10 in RNA-induced silencing complex (RISC) (Fig. 5E). Finally, qRT-PCR and western blot assays demonstrated that the RNA and protein levels of ABHD10 were negatively regulated by miR-19b-3p (Fig. 5F). Next, we validated BC002059 expression was downregulated overtly by sh-BC002059#1/#2 (Fig. 5G). In the subsequent qRT-PCR and western blot assays, we found BC002059 positively regulated ABHD10 expression (Fig. 5H). In summary, ABHD10 serves as the downstream mRNA of miR-19b-3p.

### MiR-19b-3p/ABHD10 axis affects BC002059-induced healing effects on MI

**Fig. 6:**
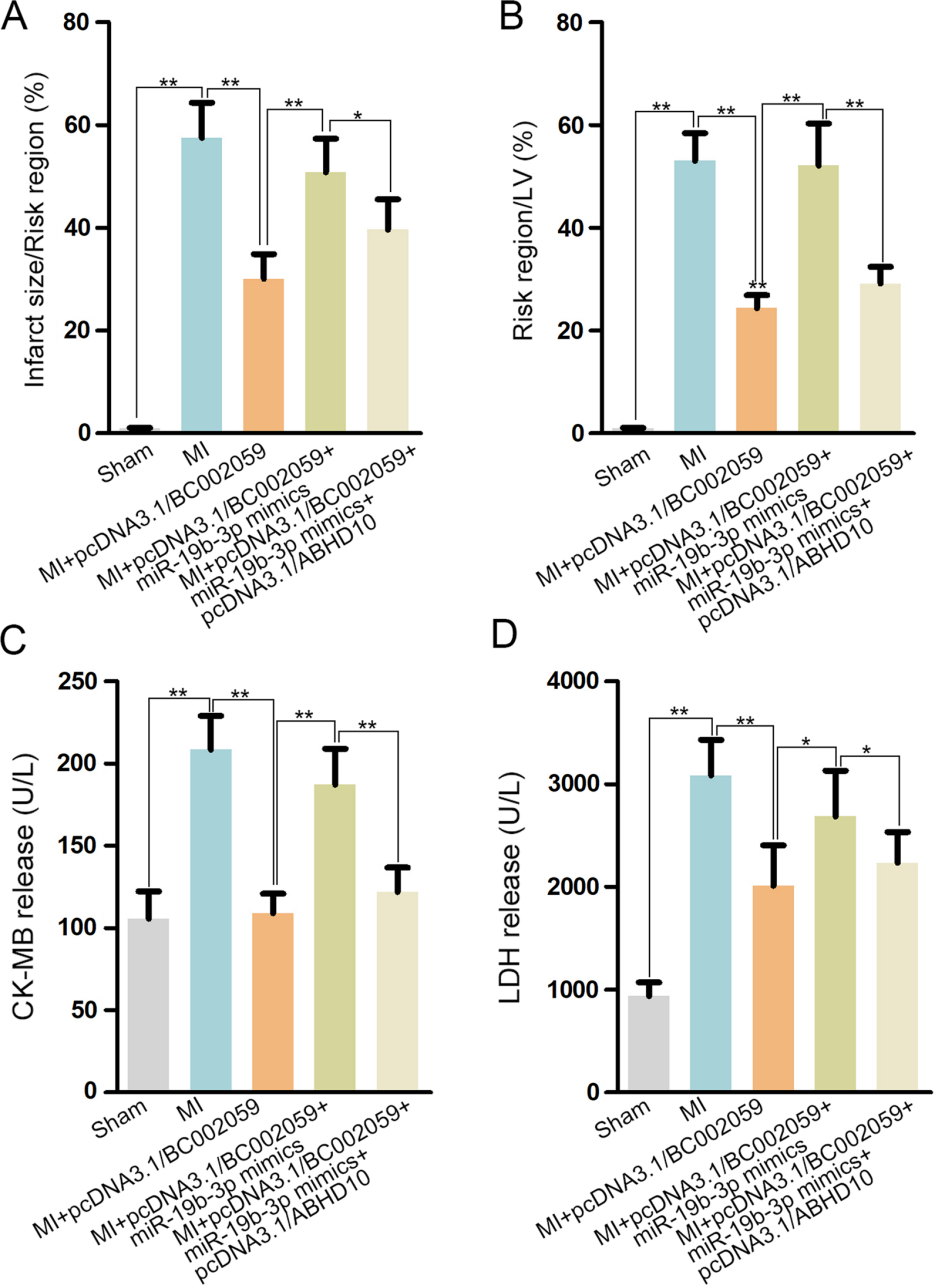
MiR-19b-3p/ABHD10 axis affects BC002059-induced healing effects on MI. **A** The percentage of infarct size/Risk region, **B** that of Risk region/LV **C** serum CK-MB release and **D** LDH release were analyzed upon different treatments. *P < 0.05, **P < 0.01

In this part, we assessed the impact of BC002059/miR-19b-3p/ABHD10 axis on MI in vivo. As expected, infarct size/risk region, risk region/LV, CK-MB and LDH release increased by MI were all reduced by BC002059 overexpression, while overexpression of miR-19b-3p countervailed this healing effects of BC002059. Moreover, ABHD10 upregulation offset the influences of miR-19b-3p mimics. The above results supported that BC002059 could protect cardiomyocytes from MI injury through regulating miR-19b-3p/ABHD10 axis (Fig. 6A–D). In sum, miR-19b-3p/ABHD10 axis affects BC002059-induced healing effects on MI.

### BC002059 modulates MI cell apoptosis via miR-19b-3p/ABHD10 axis

**Fig. 7:**
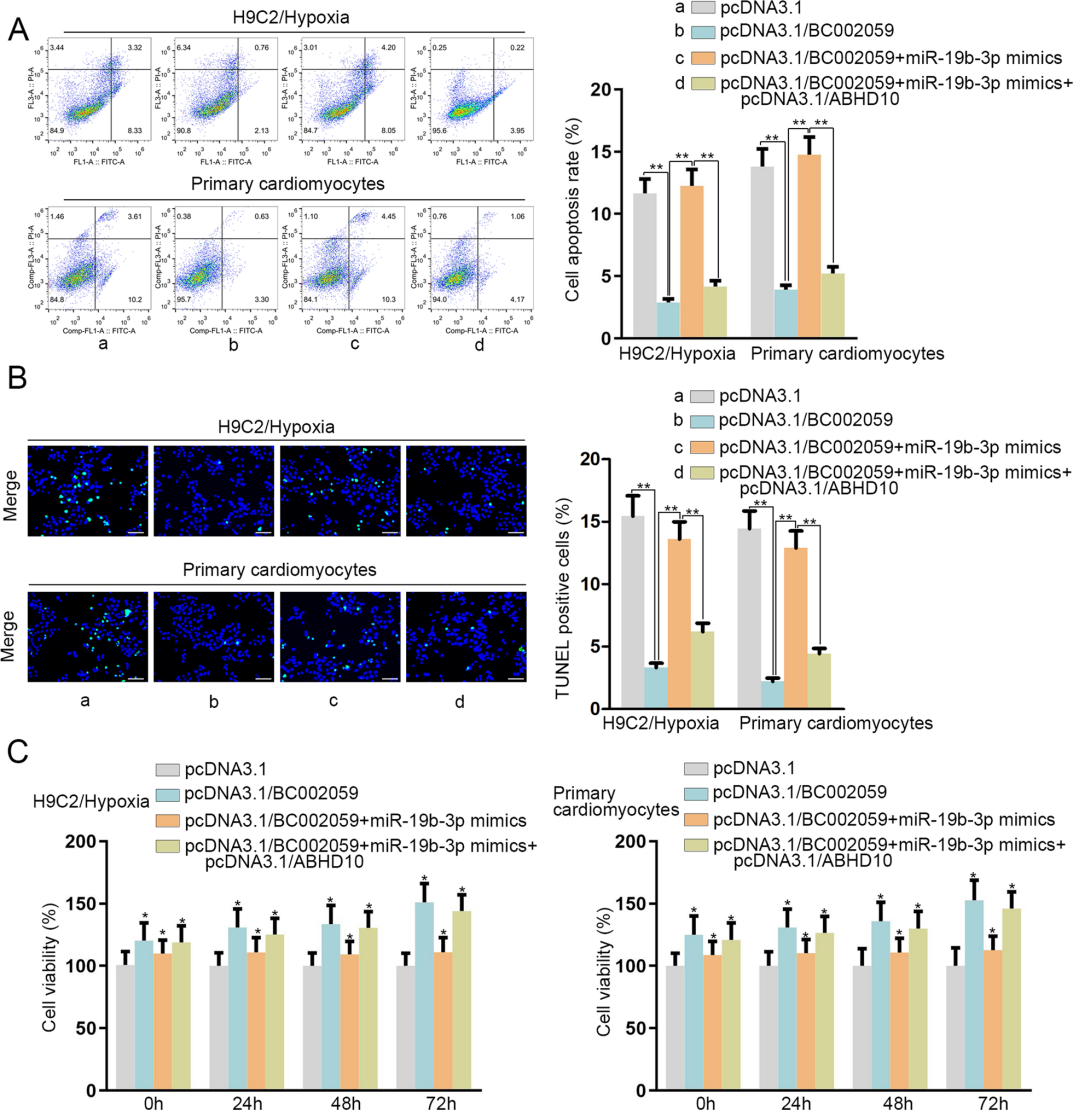
BC002059 modulates MI cell apoptosis via miR-19b-3p/ABHD10 axis. **A**, **B** Cell apoptosis was analyzed through flow cytometry and TUNEL (bar value = 1:100 μm) assays. **C** Cell viability was examined through trypan blue staining assay. *P < 0.05, **P < 0.01

To further ascertain the influence of BC002059/miR-19b-3p/ABHD10 axis on MI cells, rescue experiments were carried out. Flow cytometry analysis and TUNEL assay revealed that miR-19b-3p mimics neutralized the inhibiting effects of BC002059 overexpression on cell apoptosis rate, whereas ABHD10 upregulation counteracted the stimulating impact of miR-19b-3p mimics on cell apoptosis rate (Fig. 7A, B). Data of trypan blue staining assay suggested that pcDNA3.1/BC002059-stimulated cell viability was recovered by miR-19b-3p augment, and ABHD10 upregulation reversed the function of miR-19b-3p mimics (Fig. 7C). To sum up, BC002059 modulates MI cell apoptosis and viability via the regulation of miR-19b-3p/ABHD10 axis.

## Discussion

MI represents the most common reason leading to cardiac morbidity and mortality [21]. Accumulating evidence has demonstrated that hypothermia, as an adjuvant therapy, exert a more obvious impact for MI to decrease infarct size in addition to the poor prognosis incidence [22, 23]. Published studies have revealed crucial roles of miRNAs in influencing cell apoptosis in MI. For instance, miR-17-5p downregulation strengthens cardiac function after MI via attenuating apoptosis of endothelial cells [24]. MiR-298 modulates cardiomyocytes apoptosis after MI [25]. MiR-24 impairs cardiomyocyte apoptosis after MI by targeting with BIM [26]. In this study, the upregulated expression of miR-19b-3p in hypertrophic cardiomyopathy patients has been confirmed [14]. This study concentrated on the impact of high expression of miR-19b-3p in hypoxia-induced H9C2 cells and primary cardiomyocytes on MI cell apoptosis.

In our research, the MI mouse model was successfully constructed, which contained the larger infarct size, larger area at risk (risk region), more CK-MB/LDH release than sham group (sham-operated mice served as the control group). Based on the collected data, we observed that the myocardial cell line H9C2 and primary cardiomyocytes extracted form MI mouse model possessed the stronger apoptosis ability under hypoxia condition. Subsequently, qRT-PCR measured the obviously high expression of miR-19b-3p in MI model, H9C2 cells and primary cardiomyocytes with hypoxia treatment. Furthermore, miR-19b-3p inhibition led to the suppression of MI cell apoptosis, which verified the promoting role of miR-19b-3p in MI.

Currently, competing endogenous RNAs (ceRNAs) have been reported to play a part in post-transcriptional regulation in disease-related biological process, including MI. For example, lncRNA MALAT1 downregulation has been uncovered to relieve acute MI via modulating miR-320/Pten axis [27]. LncRNA Gpr19 deficiency weakens ischemia-reperfusion injury after acute MI through suppressing apoptosis and oxidative stress by targeting miR-324-5p/Mtfr1 axis [28]. Herein, four long non-coding RNAs (lncRNAs) that might bind with miR-19b-3p were projected through starBase. Through qRT-PCR, the expression of BC002059 was found to be significant lower in H9C2 cells and primary cardiomyocytes treated with hypoxia than other three lncRNAs. Subsequently, luciferase reporter assay confirmed the predicted binding sequence of BC002059 and miR-19b-3p. Further, gain-of-function experiments elucidated that BC002059 upregulation repressed MI cell apoptosis. Additionally, α/β hydrolase domain containing 10 (ABHD10) was verified as the target gene of miR-19b-3p in MI, and the interactions among BC002059, miR-19b-3p and ABHD10 were also confirmed due to their coexistence in RNA induced-silencing complex (RISC). Moreover, ABHD10 expression was negatively/positively modulated by miR-19b-3p/BC002059 in MI. To further validate the regulation of BC002059/miR-19b-3p/ABHD10 axis in MI cell apoptosis, rescue experiments were employed. We noticed that miR-19b-3p mimics abrogated the inhibitory impact of BC002059 overexpression in H9C2 cells and primary cardiomyocytes apoptosis as well as repressing effects on the infract region, risk region expansion, CK-MB/LDH release increase in MI mouse model. Additionally, ABHD10 increment reversed the former influence of miR-19b-3p mimics. Reviewing precious research, miR-19b has been revealed to suppress H_2_O_2_-mediated apoptosis of H9C2 cardiomyocytes through targeting PTEN [29]. Recent evidence has also suggested miR-19b attenuates MI-induced cardiac damage and induces cardiac regeneration [30]. As molecular mechanisms are complex, we suppose that different downstream genes of miR-19b-3p might lead to different impacts on cardiomyocytes. Moreover, in vivo microenvironment might account for the opposite in-vitro results in the present research.

In conclusion, our study is first to unveil the impact of BC002059/miR-19b-3p/ABHD10 axis on MI cell apoptosis, which may provide more useful information for people to understand the mechanisms in MI development. We aim to explore the upstream transcription factors and downstream signaling pathways in vivo and in vitro in the follow-up investigation to make our findings more convincing.

## Methods

### Mouse MI model

8-week-old male C57BL/6 mice (n = 56), provided by Shenzhen People’s Hospital, Second Clinical Medical College of Jinan University were applied in this research. Mouse model of MI was constructed by ligation of the left anterior descending (LAD) coronary artery. At first, 50 mg/kg ketamine and 30 mg/kg pentobarbital sodium were used to anesthetize C57BL/6 mice (n = 8). Under aseptic conditions, thoracotomy was carried out. A silk suture was utilized to ligate the left coronary artery, and then the incision was sutured. Mice (n = 8) in the Sham group underwent the same procedures except coronary artery ligation, and they were used as controls. Three days later, all mice were sacrificed and myocardial tissues were obtained. The approval of animal studies was obtained from the Animal Care and Utilization Committee of Shenzhen People’s Hospital, Second Clinical Medical College of Jinan University.

### Cell culture and treatment

DMEM medium which included 10% FBS, 3.7 g/L sodium bicarbonate, 110 mg/L sodium pyruvate and 4.5 g/L D-glucose was utilized to culture the primary cardiomyocytes (extracted from mouse MI model) and H9C2 cells (obtained from ATCC (Manassas, VA) ) at the temperature of 37 °C with 5% CO_2_. In order to simulate myocardial ischemia, H9C2 cells were subjected to anoxic treatment. Specifically, H9C2 cells were kept growing in an incubator containing 94% N_2_, 5% CO_2_, and 1% O_2_ to induce hypoxia injury [31].

### Cell transfection

Plasmids including pcDNA3.1/BC002059, sh-BC002059 (sh-BC002059#1/#2), miR-19b-3p mimics, miR-19b-3p inhibitor, pcDNA3.1/ABHD10 and their corresponding NC plasmids were all obtained from Shanghai GenePharma Co., Ltd. (Shanghai, China). Cells seeded in 24-well plates were transfected with these plasmids by Lipofectamine 2000 (Invitrogen).

### RNA extraction and qRT-PCR

TRIzol reagent was added for extracting the total RNA which was then reversely transcribed into cDNA. Subsequently, SYBR Green PCR Kit and the ABI 7500 Fast Real-Time PCR system were employed for PCR detection. The level of RNAs was calculated based on the 2^−ΔΔCt^ method. U6 or GAPDH served as endogenous control.

### Annexin V-FITC/PI double-labeled flow cytometry

On the basis of the instruction of user manual, Annexin V-FITC Apoptosis Kit was utilized to detect cell apoptosis. At first, cardiomyocytes were subjected to re-suspending in binding buffer after being digested by EDTA-free trypsin and rinsed by phosphate-buffered saline (PBS) separately. After that, Annexin V- FITC and PI were utilized to stain cells in a dark room at least 15 min at room temperature. Finally, flow cytometry was adopted to estimate cell apoptosis. The formula of cell apoptosis rate was as follows: Early and later apoptotic cell number/Total cell number.

### Nucleus and cytoplasm isolation assay

Consistent with supplier’s instruction, Nuclei Isolation Kit: Nuclei Ez Prep was used to achieve the isolation of nucleus and cytoplasm. Trizol reagent was then utilized to extract the RNAs in the separated fragments. GAPDH and U6 were used as endogenous controls.

### Luciferase reporter assay

The pmirGLO luciferase reporter plasmids which contained the sequences of BC002059-WT/ABHD10 3’UTR-WT or BC002059-Mut/ABHD10 3’UTR-Mut were synthesized. Afterwards, these plasmids were respectively transfected with miR-19b-3p mimics or NC mimics into cells. In the final step, luciferase reporter assay system was used to test the luciferase activity.

### TUNEL assay

The rTdT solution was used to cultivate the cells in a dark room at room temperature for one hour. Next, PBS and DAPI were adopted to rinse and stain the cells by turn. Eventually, with the help of a fluorescence microscope, the TUNEL-stained cells were observed.

### Trypan blue staining

Cell viability was evaluated by utilizing trypan blue staining assay kit (Beyotime, China). After transfection, primary cardiomyocytes and H9C2 cells were cultivated in a 6-well plate (1 × 10^5^ cells per well) (Thermo Fisher Scientific, USA) at 37 °C for 24 h. Subsequently, PBS and the kit solution were separately used to wash and fix the collected cells, which were counted with a microscope (Nikon, Japan). Finally, cell viability was calculated based on the formula: (the number of viable cells/the number of total cells) × 100%.

### Western blot

RIPA lysis buffer was added to extract the total proteins from the collected cells. Protein concentration was measured by BCA kit. Gel electrophoresis was used to separate the protein specimens which were then moved to the PVDF membrane. After that, the first antibodies and secondary antibodies were co-cultured with the proteins. Finally, protein bands were analyzed using ECL kit.

### Measurement of serum CK-MB and LDH

Creatine Kinase Activity Assay Kit and LDH colorimetric assay kit were utilized to estimate the serum CK-MB and LDH separately according to the protocols of suppliers. The experiment was conducted on the automatic biochemical analyzer.

### Quantification of infarct size

The tissues were sliced into two-mm thick slices. 10% formalin was used to fix tissues after they were cultivated by 2% 2, 3, 5-TTC solution for ten minutes at the temperature of 37 °C. The infarct size was measured by the use of Image-Pro Plus 6.0 software.

### RIP

As per manufacturer’s instructions, RNA Immunoprecipitation Kit was utilized to conduct RIP assay. Briefly speaking, cells were first lysed in lysis buffer. Next, lysed cells were incubated with Anti-IgG and anti-Ago2 conjugated with magnetic beads. The precipitated RNAs were isolated and quantified by qRT-PCR to assess the enrichment of target genes.

### Statistical analysis

Each independent experiment was carried out three times. Statistical analyses were conducted via SPSS18.0 statistical software package and GraphPad Prism. All quantitative data were presented in the form of mean ± standard deviation (SD). Student’s t-test, one-way or two-way ANOVA was implicated in analyzing the statistics differences between two groups or among multiple groups. Data differences represented to be statistically significant upon P < 0.05.

## Data Availability

Not applicable.

## Abbreviations

AAR: Area at risk
ABHD10: α/β hydrolase domain containing 10
ANOVA: Analysis of variance
ceRNA: Competing endogenous RNA
CK-MB: Creatine kinase-MB
DMEM: Dulbecco’s modified eagle medium
LAD: Left anterior descending
LDH: Lactate dehydrogenase
lncRNA: Long non-coding RNA
MI: Myocardial infarction
MiRNA: microRNA
mRNA: messenger RNA
Mut: Mutant
NcRNA: Non-coding RNA
PBS: Phosphate-buffered saline
PVDF: Polyvinylidene fluoride
qRT-PCR: Quantitative real-time polymerase chain reaction
RIP: RNA binding protein immunoprecipitation
RIPA: Radio immunoprecipitation assay
RISC: RNA induced-silencing complex
SD: Standard deviation
TUNEL: Terminal-deoxynucleoitidyl transferase mediated nick end labeling
WT: Wild type

## Abbreviations

AAR: Area at risk
ABHD10: α/β Hydrolase domain containing 10
ANOVA: Analysis of variance
ceRNA: Competing endogenous RNA
CK-MB: Creatine kinase-MB
DMEM: Dulbecco’s modified eagle medium
LAD: Left anterior descending
LDH: Lactate dehydrogenase
lncRNA: Long non-coding RNA
MI: Myocardial infarction
MiRNA: microRNA
mRNA: messenger RNA
Mut: Mutant
NcRNA: Non-coding RNA
PBS: Phosphate-buffered saline
PVDF: Polyvinylidene fluoride
qRT-PCR: Quantitative real-time polymerase chain reaction
RIP: RNA binding protein immunoprecipitation
RIPA: Radio immunoprecipitation assay
RISC: RNA induced-silencing complex
SD: Standard deviation
TUNEL: Terminal-deoxynucleoitidyl transferase mediated nick end labeling.
WT: Wild type
